# HOXA5 counteracts the function of pathological scar-derived fibroblasts by partially activating p53 signaling

**DOI:** 10.1038/s41419-020-03323-x

**Published:** 2021-01-05

**Authors:** Yimin Liang, Renpeng Zhou, Xiujun Fu, Chen Wang, Danru Wang

**Affiliations:** Department of Plastic and Reconstructive Surgery, Shanghai 9th People’s Hospital, Shanghai Jiao Tong University School of Medicine, 200011 Shanghai, China

**Keywords:** Cell growth, Cell signalling, Senescence

## Abstract

The inactivation of p53 can lead to the formation of pathological scars, including hypertrophic scars and keloids. HOXA5 has been reported to be a critical transcription factor in the p53 pathway in cancers. However, whether HOXA5 also plays a role in pathological scar progression through activating p53 signaling remains unknown. In this study, we first demonstrated that HOXA5 overexpression in hypertrophic scar-or keloids-derived fibroblasts decreased cell proliferation, migration and collagen synthesis, whereas increased cell apoptosis. Furthermore, the results of luciferase activity assays and ChIP PCR assays indicated that HOXA5 transactivated p53 by binding to the ATTA-rich core motif in the p53 promoter. HOXA5 also increased the levels of p21 and Mdm2, which are downstream targets of p53. Interestingly, silencing p53 in these pathological scar-derived fibroblasts partially attenuated HOXA5-mediated growth inhibition effect and HOXA5-induced apoptosis. In addition, 9-cis-retinoic acid augmented the expression of HOXA5 and promoted the effects of HOXA5 on pathological scar-derived fibroblasts, and these effects could be suppressed by HOXA5 knockdown. Thus, our study reveals a role of HOXA5 in mediating the cellular processes of pathological scar-derived fibroblasts by transcriptionally activating the p53 signaling pathway, and 9-cis-retinoic acid may be a potential therapy for pathological scars.

## Introduction

Pathological scars, including keloids and hypertrophic scars that are characteristic of excessive dermal fibrosis, are caused by excessive cell proliferation and collagen synthesis during the wound healing process. Pathological scars can result in severe organ malformation and dysfunction and are consistently difficult to cure. Therefore, the underlying molecular mechanism of scar formation and the anti-scarring strategy are the research hotspots.

The wound healing process includes three overlapping but distinct stages: inflammation, granulation tissue formation, and remodeling^1^. Each group of stage-specific cells sequentially occur at the wounded site, proliferate, and then quit after the tasks have been completed. Apoptosis is confirmed to be the primary mechanism for cells quit^2^. Previous studies have demonstrated that pathological scars usually form due to defective apoptotic signals that lead to fibroblast dysfunction^3–5^. The p53 tumor suppressor plays a crucial role in regulating apoptosis^6,7^. However, the molecular mechanisms by which p53 participates in pathological scarring remain unclear and controversial.

In human breast cancer, the loss of p53 expression may be caused by a lack of homeobox A5 (HOXA5) expression^8^. Homeobox genes are a group of genes that play key roles in both development and postnatal regeneration^9^. Our previous study revealed that HOXA5 was able to suppress keratinocyte growth and epidermal formation^10^, we also showed that HOXA5 was substantially activated by mechanical stretch in renewed skin, suggesting a role for HOXA5 in regulating skin regeneration, including scarless wound healing^11^. While HOXA5 can promote breast cancer cell death through a p53-dependent apoptotic pathway^8^, the mechanism by which HOXA5 transcriptionally regulates p53 in pathological scars remains unknown.

Here, we hypothesized that HOXA5 could inhibit the pathological scarring by activating the p53 pathway. Therefore, we investigated the expression and role of HOXA5 in the cellular processes of pathological scar-derived fibroblasts, both from keloids (KFb) and hypertrophic scars (HSFb), as well as the underlying molecular mechanism by which HOXA5 regulates the p53 pathway. In addition, the effects of 9-cis-retinoic acid on HOXA5 expression and KFb and HSFb cellular processes were examined to identify a potential therapy to prevent scarring.

## Materials and methods

### Tissue collection and fibroblasts culture

Normal primary fibroblasts (Fb) were isolated and cultured from the normal skin of patients who underwent abdominoplasty. Written consent was obtained. Primary fibroblasts (HSFb and KFb) were isolated and cultured from the hypertrophic scars and keloids, respectively, of 15 patients after obtaining informed consent of the patients and approval of the Shanghai ninth hospital’s Ethical Committee. The mean patient age was 32.7 years (range 22–45 years) and there were nine women and six men. Briefly, the harvested tissue samples were washed with phosphate-buffered saline, cut into pieces and digested with 0.3% collagenase type I (Serva,Germany). The digested tissue samples were filtered through a 200-µm mesh filter, and centrifuged at 500 × *g* for 10 min. The supernatant was discarded, and the pellet was resuspended in DMEM (HyClone, USA) and cultured in DMEM supplemented with 10% fetal bovine serum (Hyclone, USA) in 5% CO_2_ at 37 °C. Primary cells at passages 2–3 were used for the experiments.

### Expression vector construction and transfection procedures

The sequence of HOXA5 was amplified from wild-type complementary DNA (cDNA) by PCR. The following primer sequences were used: forward: 5ʹ-GAGGATCCCCGGGTACCGGTCGCCACCATGAGCTCTTATTTTGTAAAC-3ʹ; reverse: 5ʹ-TCACCATGGTGGCGACCGGGGGACGGAAGGCCCCTCCTG-3ʹ. The HOXA5 PCR fragments and the pGC-FU vector (Shanghai GeneChem,China) were digested with Age I and then ligated with T4 DNA ligase to produce the pGC-FU-HOXA5. The empty pGC-FU vector was used as the control vector. 293T cells were transfected with pGC-FU-HOXA5, pHelper 1.0 and pHelper 2.0 (Shanghai GeneChem, China) to generate the viral particles. Then fibroblasts were plated into six-well plates, cultured with 10% fetal bovine serum/Dulbecco’s modified Eagle’s medium (DMEM) for 24 h and then transfected with supernatants containing the lentivirus.

### CCK-8 assay

The cell proliferation of fibroblasts was measured by the Cell Counting Kit-8 assay (CCK-8 assay, Sigma) according to the manufacturer’s instructions. At 24 h posttransfection with HOXA5, the fibroblasts were seeded in 96-well plates at 3000 cells/well. The absorbance was measured at 450 nm at the indicated time points.

### Cell viability and apoptosis analysis

Cell viability and apoptosis were detected using the Annexin V-FITC Apoptosis Detection Kit (Sigma, APOAF). HSFb and KFb were harvested, centrifuged at 300 × *g* and resuspended in binding buffer. Then 5 μl Annexin V-FITC and 10 μl propidium iodide solution were added to each cell suspension and incubated in the dark. The samples were kept on ice and analyzed immediately analyzed by flow cytometry with a FACSCalibur instrument (BD Biosciences, USA). The data were further analyzed with FlowJo software (FlowJo, OR) to calculate the proportion of apoptotic cells.

### Transwell assay

HSFb and KFb were digested with 0.25% trypsin and suspended in serum-free medium, 1 × 10^4^ cells were seeded in a Matrigel-coated Transwell chamber (8-µm pore,size,Corning). Complete medium (500 µl) was added to the lower chamber. After incubation for 24 h, the nonmigrated fibroblasts were removed from the upper surface of the membrane. The migrated fibroblasts were fixed in 4% paraformaldehyde and stained with 0.4% crystal violet solution (Beyotime, China). The migrated fibroblasts were visualized under an inverted microscope (Olympus, Japan), and the average numbers of fibroblasts were calculated by quantifying the cells in ten randomly selected fields.

### Fibroblast-populated collagen lattice (FPCL)

Rat tail type I collagen (Gibco,USA) solutions were mixed with HSFb or KFb at a density of 3 × 10^5^/ml. The mixture was seeded into the 24-well plates and then incubated at 37 °C. The collagen gels were imaged after 48 h and the degree of gel contraction was determined by calculating the ratio of the final contracted gel areas to the initial gel areas.

### Western blot

HSFb and KFb were lysed with RIPA buffer (Beyotime, China) and the supernatants were harvested. The 25 μg protein samples were separated by 10% sodium dodecyl sulfate polyacrylamide gel electrophoresis and transferred to polyvinylidene fluoride membranes. The membrane was blocked for 1 h at room temperature and incubated with primary antibodies overnight at 4 °C. The antibodies used were as follows: mouse anti-GAPDH(1:2000, Abcam) mouse anti-α-SMA (1:500, Abcam), mouse anti-vinculin (1:500, Abcam), rabbit anti-collagen I (1:500, Abcam), rabbit anti-collagen III (1:500, Abcam), mouse anti-p21 (1:500, Abcam), mouse anti-Mdm2 (1:500, Abcam), Rabbit anti-HOXA5(1:1000, Acam), mouse anti-p53(1:1000, Abcam). The membranes were washed three times with PBST and incubated with the corresponding HRP-conjugated secondary antibodies for 1 h at room temperature. The protein levels were detected using an ECL reagent kit (Thermo Fisher, USA). The ChemiDoc MP imaging system (Bio-Rad, USA) was used to detect the protein expression. The data were analyzed using ImageJ software (NIH, USA). GAPDH was used as the endogenous reference for normalization.

### Quantitative real-time PCR

Total RNA was extracted using TRIzol and the RNA concentration was determined using a NanoDrop2000 spectrophotometer (Thermo Scientific). Then 500 ng RNA was used to synthesize cDNA with reverse transcriptase (Takara) and the messenger RNAs (mRNAs) levels were quantified using the GoTaq qPCR Master Mix(Promega, USA). The sequences of primers were as follows: GAPDH forward: GCACCGTCAAGGCTGAGAAC, GAPDH reverse: TGGTGAAGACGCCAGTGGA, α-SMA forward: AGGTAACGAGTCAGAGCTTTGGC, α-SMA reverse: CTCTCTGTCCACCTTCCAGCAG, vinculin forward: AGAGACTGTTCAGACCACTGAG, vinculin reverse: CATTGAGTTCACCAACATCAC, Col1A1 forward: AAGAGCTCGTGGGAAAGCCTGGATGG, Col1A1 reverse: AAAGATCTTTTGGGACTTACTGTCTTCGT, Col3A1 forward: CCCAGAACATCACATATCAC, Col3A1 reverse: CAAGAGGAACACATATGGAG. Mdm2 forward: GAATCATCGGACTCAGGTACATC, Mdm2 reverse: TCTGTCTCACTAATTGCTCTCCT; p53 forward: CCTCAGCATCTTATCCGAGTGG, reverse:TGGATGGTGGTACAGTCAGAGC; p21 forward: AGGTGGACCTGGAGACTCTCAG, reverse: TCCTCTTGGAGAAGATCAGCCG. The reactions were performed in triplicate. The mRNA levels were normalized to that of GAPDH by using the delta-delta Ct method.

### Luciferase reporter assay

A luciferase reporter construct containing the p53 promoter was purchased from Shanghai Gema. Before transfection, HSFb or KFb were seeded into 24-well plates. The fibroblasts were transfected with the luciferase promoter constructs and either a HOXA5 overexpression construct or an empty vector. The cells were lysed at 24 h after transfection and the luciferase activity was measured using the luciferase reporter assay system (Promega) according to the manufacturer’s instructions.

### Chromatin immunoprecipitation (ChIP) PCR

HSFb and KFb in the HOXA5-overexpressing group or control group were crosslinked in 1% formaldehyde for 10 min at room temperature. The fibroblasts were lysed and sonicated to obtain 200–500 bp genomic DNA fragments. The sheared fragments were incubated overnight at 4 °C with rabbit anti-HOXA5 or with control rabbit IgG. After blocking in 0.5% BSA, the Dynabeads (Invitrogen) were added to couple the antibodies. The precipitated DNA fragments were purified and used for PCR analysis. Primers designed to analyze the binding of the p53 promoter were as follows: forward TGCTCAAGACTGGCGCTAAA; reverse GGAGCTTACCCAATCCAGGG.

### Small-interfering RNA (siRNA) transfection

Synthetic siRNA targeting human HOXA5 or p53 and nonspecific control siRNA were purchased from GeneChem (Shanghai, China). Fibroblasts were seeded into 6-well plates and cultured to 60% confluence and then transfected with HOXA5 siRNA or p53 siRNA or control siRNAs. At 48 h after transfection, the cells were harvested for qPCR detection and 72 h after transfection, the cells were harvested for western blotting analysis.

### 9-cis-retinoic acid(9-cis-RA) treatment

For the treatment of HBFb and KFb, 9-cis-retinoic acid (9-cis-RA, Sigma) was dissolved in dimethyl sulfoxide. Cultured HSFb and KFb were treated with 0.1 µmol/l 9-cis-RA, and the cells in the control group were treated with standard medium.

### Statistical analyses

All the experiments were repeated three times. The data were analyzed by SPSS12.0. One-way analysis of variance (ANOVA) test was used to identify significant differences among experimental groups. The *t*-test was performed to identify statistically significant differences between two groups. The results are expressed as the mean ± standard deviation.

## Results

### Effects of HOXA5 overexpression on pathological scar-derived fibroblasts

**Fig. 1:**
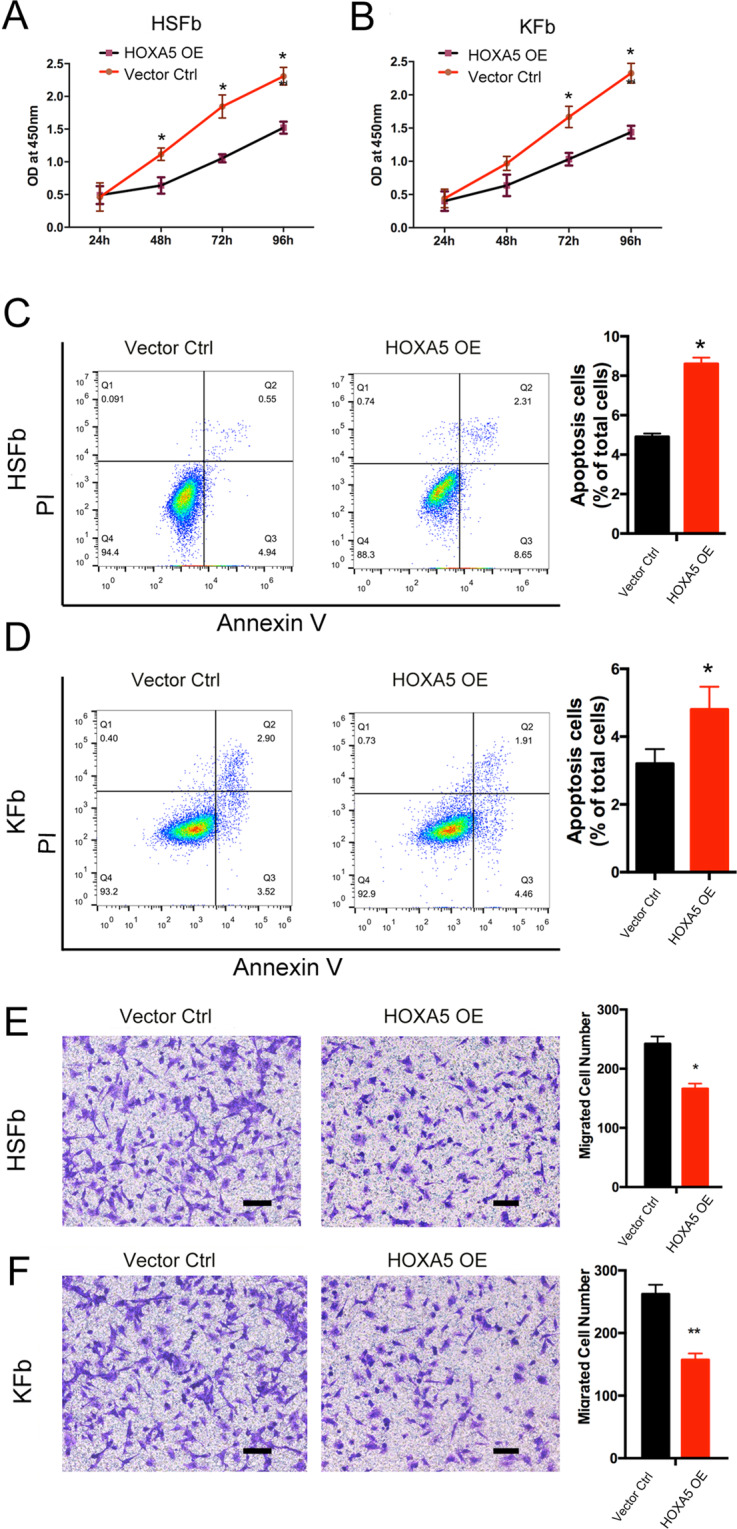
Effects of HOXA5 overexpression on pathological scar-derived fibroblasts. **A**, **B** A CCK-8 assay was performed to determine the cell proliferation rates of fibroblasts derived from keloids (KFb) or from hypertrophic scars (HSFb) with/without HOXA5 overexpression. **C**, **D** Flow cytometric analysis was performed to determine the percentages of apoptotic KFb and HSFb in each group. **E**, **F** Transwell analysis was performed to determine the cell migration ability of KFb and HSFb in each group. *p < 0.05, **p < 0.01. scale bar = 100 μm.

**Fig. 2:**
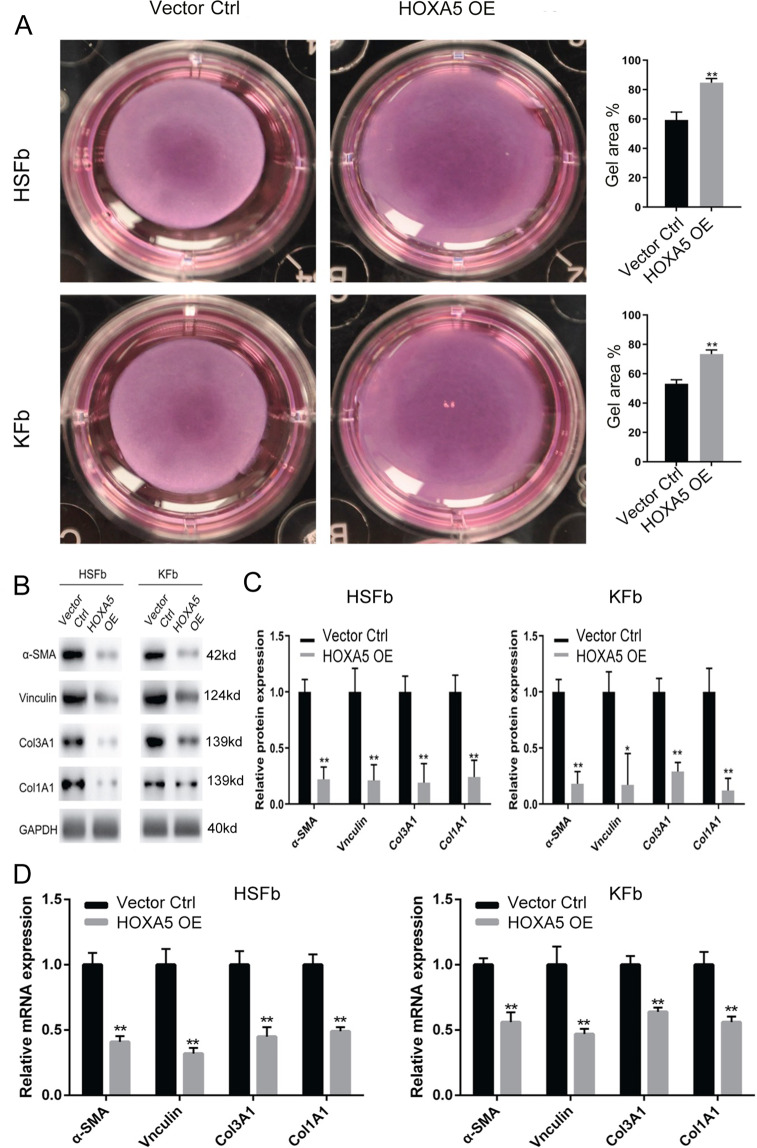
Role of HOXA5 in pathological scar-derived fibroblasts. **A** A fibroblast-populated collagen lattice was used to measure the contractive abilities of KFb and HSFb in each group. **B**–**D** The expression of fibroproliferative biomarkers, including α-SMA, Vinculin, ColI, and Col III in KFb and HSFb in each group was detected by western blot and qPCR analysis. *p < 0.05, **p < 0.01.

To investigate the role of HOXA5 in the cell biology of fibroblasts derived from keloids (KFb) or from hypertrophic scars (HSFb), we established HOXA5 overexpression in both types of scar-derived fibroblasts (Fig. S1). The CCK-8 assay showed that compared with the control (Vector Ctrl), HOXA5 overexpression (HOXA5 OE) significantly inhibited the cell proliferation of HSFb at 48 (1.12 ± 0.09 vs. 0.63 ± 0.12), 72 (1.85 ± 0.17 vs. 1.05 ± 0.06) and 96 (2.31 ± 0.13 vs. 1.52 ± 0.09) hours (Fig. 1A) and decreased the KFb cell proliferation at 72 (1.66 ± 0.16 vs. 1.03 ± 0.09) and 96 (2.53 ± 0.15 vs. 1.43 ± 0.09) hours (Fig. 1B). We next investigated the effects of the HOXA5 overexpression on the apoptosis of KFb and HSFb by flow cytometry. As shown in Fig. 1C, D, compared with the percentage of apoptotic HSFb and KFb in the control group (HSFb: 4.9 ± 0.2%; KFb:3.2 ± 0.4%), the percentage of apoptotic cells in the HOXA5 overexpression group was significantly higher (HSFb:8.6 ± 0.3%; KFb:4.8 ± 0.6%). We also performed the transwell assay to investigate the potential function of HOXA5 in the migration of scar-derived fibroblasts. Compared with the number of migrated KFb and HSFb in the control group (HSFb:262 ± 15; KFb:242 ± 12), the number of migrated KFb and HSFb in the HOXA5 overexpression group(HSFb:166 ± 8; KFb:157 ± 10) was significantly reduced (Fig.1E, F). Then, we further measured the effects of HOXA5 on cell contractive ability using a fibroblast-populated collagen lattice (FPCL). The data showed that the KFb and HSFb in the HOXA5 overexpression group exhibited impaired cell contractive ability (Fig. 2A). We also performed qPCR and western blot assays to investigate the effects of HOXA5 on extracellular matrix secretion, and the data showed that the HOXA5 overexpression decreased the expression of α-SMA, Vinculin, ColI and Col III (Fig. 2B–D). In addition, when compared with the control group, the HOXA5 knockdow group exhibited significantly increased protein level of α-SMA, Vinculin, ColI and Col III (Fig. S2).

### HOXA5 binds to the p53 promoter and activates p53 signaling in both HSFb and KFb

**Fig. 3:**
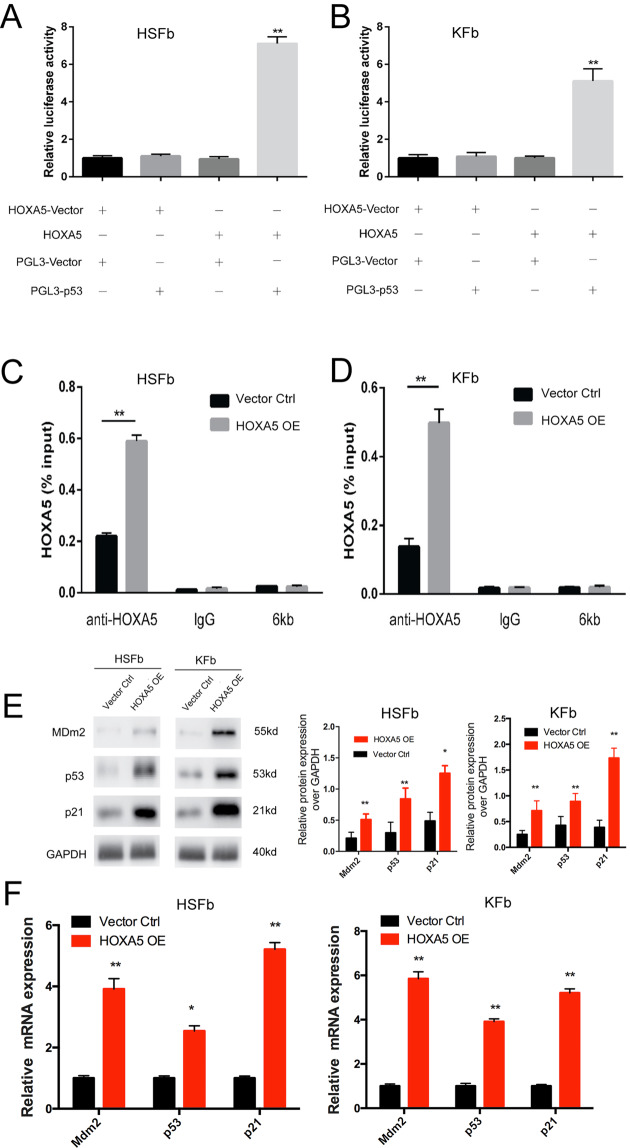
HOXA5 binds to the p53 promoter and activates p53 signaling in both HSFb and KFb. **A**, **B** Luciferase assay data showed that HOXA5 activated the expression of p53 promoter reporter gene in KFb and HSFb. **C**, **D** ChIP data showed a direct interaction between HOXA5 and p53. 6 kb:the 6 kb upstream of the transcription start site, was used as a negative control for the transcription start site. **E**, **F** qPCR and western blot data showed that the overexpression of HOXA5 enhanced the expression of p21 and Mdm2, which are downstream targets of p53. *p < 0.05, **p < 0.01.

To investigate whether HOXA5 affects pathological phenotypes by regulating p53 expression, we co-transfected both the HOXA5-overexpressing plasmid and the p53 promoter luciferase reporter construct (the initiation of transcription is at the 2 kb upstream) into both HSFb and KFb, and then detected the luciferase activity. The data showed that HOXA5 was able to activate the expression of the p53 promoter reporter gene in both kinds of cells (Fig. 3A, B). To confirm a direct interaction between HOXA5 and p53, we further detected the binding of HOXA5 and the ATTA-rich core motif in the p53 promoter using chromatin immunoprecipitation (ChIP) coupled with quantitative PCR. We found that after the overexpression of HOXA5, the level of binding was enhanced (Fig. 3C, D). Furthermore, both the mRNA expression and the protein levels of p21 and Mdm2, which are the downstream targets of p53, were significantly upregulated in both kinds of fibroblasts in the HOXA5 overexpression group (Fig. 3E, F). Taken together, these data suggested that HOXA5 may affect pathological phenotypes by transcriptionally upregulating the expression of p53.

### Knockdown of p53 partially weakened HOXA5-mediated regulation in both HSFb and KFb

**Fig. 4:**
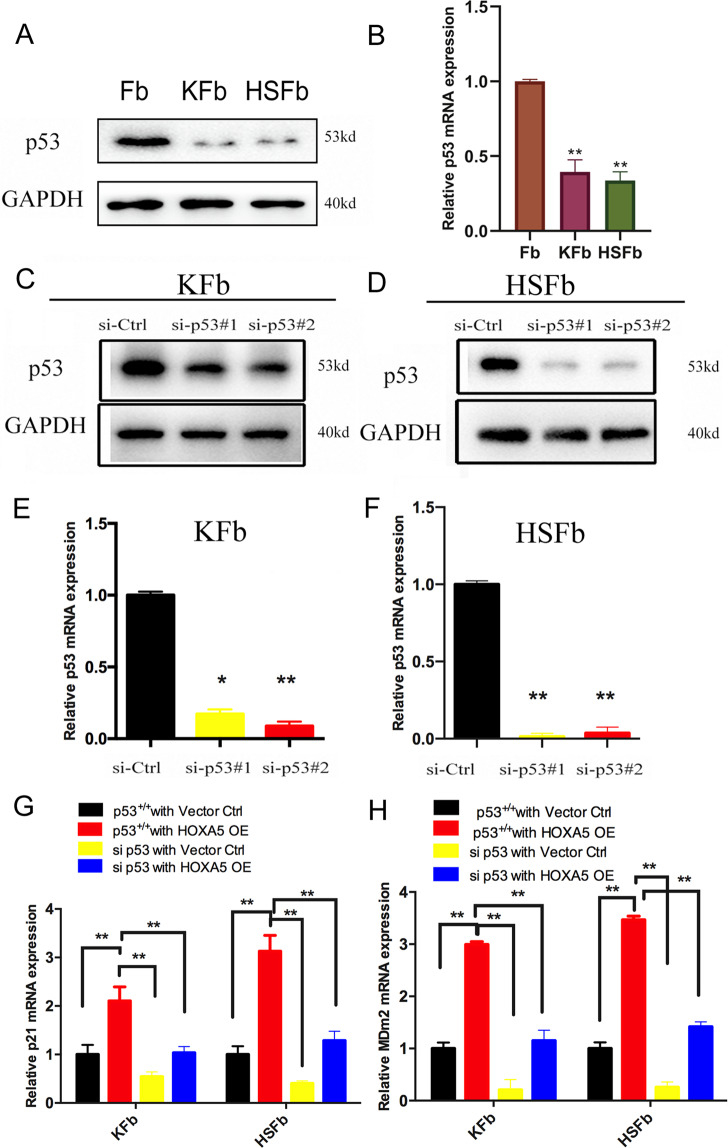
Knockdown of p53 in both the HSFb and the KFb. **A**, **B** The expression of p53 in KFb, HSFb and normal fibroblasts was detected by qPCR and western blot. **C**, **D** p53 protein levels in KFb and HSFb were detected by western blot. **E**, **F** p53 mRNA levels were detected by QPCR in KFb and HSFb. **G**, **H** Knockdown of p53 partially weakened the HOXA5-mediated regulation of p21 and Mdm2 in KFb and HSFb. *p < 0.05, **p < 0.01.

To investigate the potential mechanism underlying HOXA5-mediated p53 upregulation in HSFb and KFb, we further analyzed p53. The qPCR and western blot analyses showed that the expression of p53 was significantly lower in KFb and HSFb than in normal fibroblasts (Fig. 4A, B). To determine whether p53 facilitated the HOXA5-mediated regulation on both the KFb and the HSFb, we knocked down (KD) p53 using two siRNAs targeting p53. The kockdown efficiency was confirmed by qPCR and western blot (Fig. 4C–F).

To further elucidate whether p53 contributes to HOXA5-mediated phenotypes in pathological scar-derived fibroblasts, we conducted reconstitution experiments by transfecting HSFb and KFb with si-p53 or the HOXA5 overexpression plasmid alone or with si-p53 and the HOXA5 overexpression plasmid in combination. Compared with control (p53^+/+^ with vector ctrl group), HOXA5 overexpression alone (p53^+/+^ with HOXA5 OE group) promoted the expression of p21 and Mdm2 in both HSFb and KFb. However, this effect was attenuated by the knockdown of p53 (si p53 with HOXA5 OE group) (Fig. 4G, H).

**Fig. 5:**
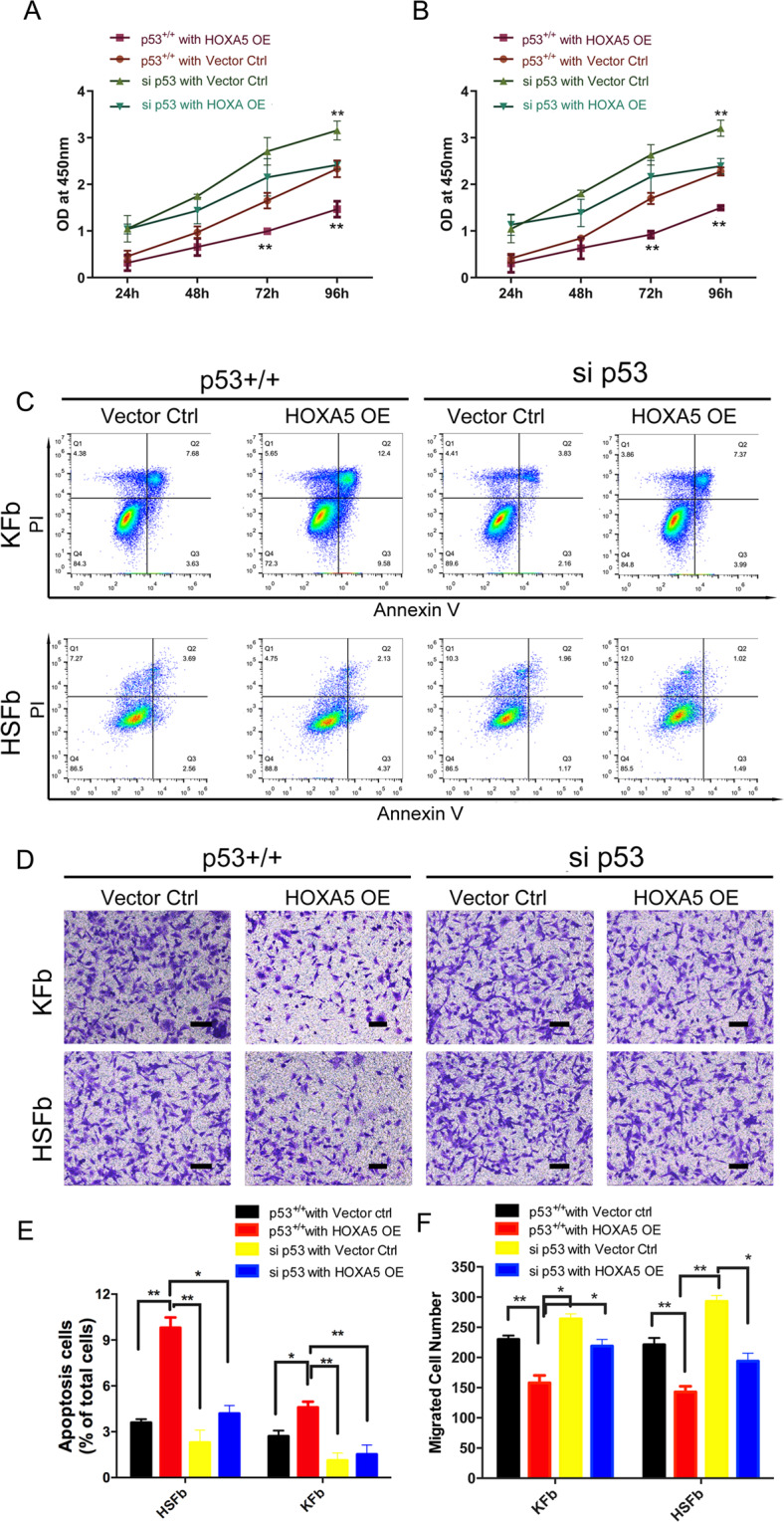
Knockdown of p53 partially weakens HOXA5-mediated regulation in both HSFb and KFb. **A**, **B** Knockdown of p53 partially weakened the HOXA5-mediated regulation of cell proliferation in KFb and HSFb. **C**, **E** Knockdown of p53 partially weakened the HOXA5-mediated regulation of cell apoptosis in KFb and HSFb. **D**, **F** Knockdown of p53 partially weakened -mediated regulation of cell migration in KFb and HSFb. *p < 0.05, **p < 0.01. scale bar = 100 μm.

Then, we performed proliferation, migration and apoptosis assays. Compared with control (p53^+/+^ with vector ctrl group), HOXA5 overexpression alone (p53^+/+^ with HOXA5 OE group) inhibited cell proliferation in both HSFb and KFb. However, this effect was attenuated by p53 knockdown (si p53 with HOXA5 OE group), indicating a critical role of p53 in the HOXA5-mediated inhibition of proliferation (Fig. 5A, B). Similarly, in the cellular apoptosis analyses with Annexin V-FITC/PI double staining, HOXA5 (HSFb:9.8 ± 0.3%; KFb:4.6 ± 0.2% in p53 + /+ with HOXA5 OE group) induced cell apoptosis in both HSFb and KFb compared with the control group(HSFb:3.6 ± 0.2%; KFb:2.7 ± 0.3% in p53 + /+ with vector ctrl group), and si-p53 (HSFb:4.2 ± 0.4%; KFb:1.5 ± 0.2% in si p53 with HOXA5 OE group)partially abolished the HOXA5-mediated cell apoptosis (Fig. 5C, E). Moreover, HOXA5 overexpression alone (HSFb:143 ± 9; KFb:158 ± 12) inhibited the cell migration of both HSFb and KFb compared with control (HSFb:221 ± 11; KFb:230 ± 6), and this effect was attenuated when p53 was knocked down (HSFb:194 ± 13; KFb:219 ± 11) (Fig. 5D, F). These results indicated that HOXA5 regulated the cell function of HSFb and KFb, at least partially by regulating p53.

### 9-cis-retinoic acid increases HOXA5 expression and promotes HOXA5-mediated effects on HSFb and KFb

**Fig. 6:**
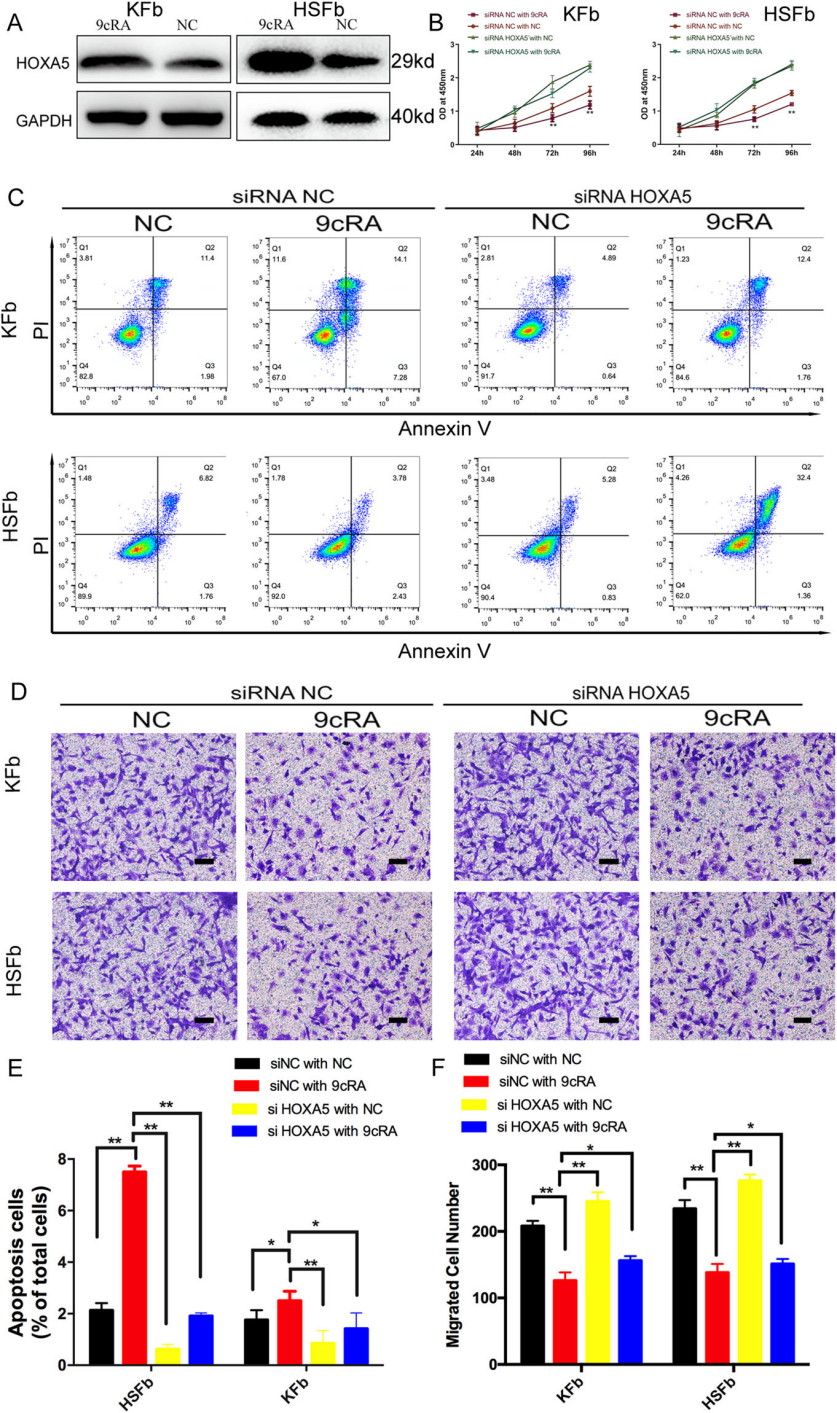
9-cis-retinoic acid increases HOXA5 expression and promotes HOXA5-mediated effects on HSFb and KFb. **A** 9-cis-retinoic acid (9cRA) enhanced the expression of HOXA5 in KFb and HSFb. **B** Knockdown of HOXA5 inhibited the 9cRA-mediated regulation of cell proliferation. **C**, **E** Knockdown of HOXA5 inhibited the 9cRA-mediated regulation of cell migration in KFb and HSFb. **D**, **F** Knockdown of HOXA5 inhibited the 9cRA-mediated regulation of cell apoptosis in KFb and HSFb. *p < 0.05, **p < 0.01. scale bar = 100 μm.

Retinoic acid is known to stimulate the expression of HOXA5 in some cancer cells and has been evaluated as a preventive and therapeutic agent^12–14^. To test whether the 9-cis-retinoic acid (9cRA) is an agent that could potentially be used for the treatment of pathological scars, HSFb and KFb were treated with 9cRA alone, knockdown HOXA5 alone or 9cRA and HOXA5 in combination. The data showed that the expression of HOXA5 in pathological scar-derived fibroblasts significantly increased when the cells were treated with 9cRA (Fig. 6A). Then we established and confirmed the HOXA5 knockdown in both scar-derived fibroblasts (Fig. S3). 9cRA treatment alone (siRNA NC with 9cRA group) inhibited cell proliferation compared with the control (siRNA NC with NC group) in both HSFb and KFb. However, this effect was attenuated upon knockdown of HOXA5 (si-HOXA5 with 9cRA group), indicating a critical role of HOXA5 in the 9cRA-mediated inhibition of proliferation (Fig. 6B). Similarity, in cellular apoptosis assay, 9cRA treatment (HSFb:7.5 ± 0.2%; KFb:2.5 ± 0.1% in the siRNA NC with 9cRA group) induced cell apoptosis in both HSFb and KFb compared with the control group (HSFb:2.1 ± 0.1%; KFb:1.76 ± 0.1% in siRNA NC with NC group), and HOXA5 knockdown (HSFb:1.9 ± 0.3%; KFb:1.4 ± 0.3% in si-HOXA5 with 9cRA group) attenuated the 9cRA-induced cell apoptosis (Fig. 6C, E). Moreover, 9cRA treatment alone(HSFb:138 ± 13; KFb:126 ± 10) inhibited the cell migration of both HSFb and KFb compared with the control (HSFb:234 ± 15; KFb:208 ± 7), and this effect was attenuated when HOXA5 was knocked down (HSFb:151 ± 7; KFb:156 ± 6) (Fig. 6D, F). The results indicated that HOXA5 is a key effector of 9-cis-retinoic acid.

## Discussion

Our data revealed that the overexpression of HOXA5 in both kinds of fibroblasts decreased proliferation, migration and collagen synthesis and increased cell apoptosis. The gain/loss-of-function studies showed that HOXA5 was the upstream regulator responsible for the aberrant expression of p53 during scar formation and progression. Moreover, HOXA5 transcriptionally activated p53 and its downstream targets (p21 and Mdm2) by regulating binding to the p53 promoter. In addition, 9-cis-retinoic acid induced the expression of HOXA5, while knockdown of HOXA5 abolished the function of 9-cis- retinoic acid in both KFb and HSFb. Therefore, these findings confirmed the importance of the HOXA5-p53 signaling pathway in pathological scar progression and indicated a potential role of 9-cis-retinoic acid in therapeutic strategies to prevent scarring.

Hypertrophic scars and keloids are fibrotic diseases characterized by aberrant fibroblast proliferation and collagen deposition. The formation of these two pathological scars involves many factors, and the etiology remains unclear. Treatment aimed at molecular targets is considered as a promising option^15,16^. Here, we tried to investigate the effect of p53 on pathological scars, as well as the underlying mechanism.

P53 is widely known as a transcription factor that controls cell proliferation and apoptosis. However, the expression and role of p53 in hypertrophic scars and keloids remain controversial. Ladin et al.^17^ showed that the p53 is significantly upregulated in keloids and keloid-derived fibroblasts compared with normal tissue. Tanaka et al.^18^ also reported that the expression of p53 is significantly higher in keloids and hypertrophic scars than in normal skin. On the other hand, low levels, and even no expression, of p53 were detected in hypertrophic scars and keloids in several previous studies^6,19^. Previous studies also showed that the overexpression of p53 in HSFb promoted autophagy and inhibited collagen expression^7^, while p53 knockout promoted hypertrophic scar formation^20^. Here, we showed that p53 is significantly downregulated in both HSFb and KFb, and we further demonstrated that knockdown of p53 promoted cell proliferation, migration and inhibited apoptosis in HSFb and KFb. It was reported that 5-fluorouracil (5-Fu) can induce the accumulation of p53 in keloids^21^, and the combination of topical 5-Fu with steroids is a treatment strategy for hypertrophic scars and keloids in clinic^22,23^. Although further investigation is needed, it is interesting to hypothesize that pathological scar tissues that previously received 5-Fu treatment may have a higher expression of p53. In addition, although keloids and hypertrophic scars have common characteristics including excessive collagen accumulation, keloids tend to extend beyond the site of injury while hypertrophic scars tend to regress. The different roles of the 9-cis-RA/HOXA5/P53 pathway in each fibrotic condition require further investigation.

HOXA5 plays a role in activating the p53-dependent pathway in several cancers^8,9^, however, the expression of HOXA5 and its molecular mechanism in hypertrophic scars or keloids have not been investigated. Our data demonstrated that HOXA5 is a positive regulator of p53 transcription and function in cultured fibroblasts derived from pathological scars, although its function is not completely dependent on p53. In breast cancer cells, it has been shown that HOXA5 can induce cell apoptosis through a p53-dependent^8^ and p53-independent apoptotic pathway^24^. Thus, whether HOXA5 induces apoptosis through alternative pathways in keloids and hypertrophic scars requires further investigation.

Retinoic acid has been used as a therapeutic agent for a variety of cancers and HOXA5 is a critical mediator of this effect^14,25^. In contrast to a previous study claimed that HOXA5 knockdown only partially inhibited RA-induced apoptosis in breast cancer cells^14^, our data showed that 9-cis-retinoic acid transfection could strongly induce HOXA5 expression and inhibit cell proliferation and migration. Moreover, the knockdown of HOXA5 completely inhibited the function of the 9- cis-retinoic acid

In addition, an important finding of our present work was that HOXA5 regulated the expression of the fibrosis-related genes, Col1A1,Col3A1 and vinculin in fibroblasts via the p53 pathway.

In conclusion, our results revealed a key role of HOXA5 in the inhibition of pathological scarring through the p53 pathway and proved that 9cRA is a promising candidate for clinical treatment strategies.

## Supplementary information

Supplemental figure legends

Supplemental figure 1

Supplemental figure 2

Supplemental figure 3
