# Supplementary material for: HOXA5 counteracts the function of pathological scar-derived fibroblasts by partially activating p53 signaling
Source: Cell Death Dis. 2021 Jan 5;12(1):40. doi: 10.1038/s41419-020-03323-x (PMC7791133; doi:10.1038/s41419-020-03323-x)
Supplement: Supplementary file 1 — Supplemental figure legends [file 41419_2020_3323_MOESM1_ESM.docx]

Supplementary figure legends

Supplemental figure 1: HOXA5 protein levels were detected after overexpression of HOXA5 in HSFb and KFb.

Supplemental figure 2: HOXA5 protein levels were detected after knock down of HOXA5 in HSFb and KFb.

Supplemental figure 3: The expression of α-SMA, Vinculin, ColI and Col III was up-regulated following knockdown of HOXA5 in HSFb and KFb.
