# Supplementary figures and images for: HOXA5 counteracts the function of pathological scar-derived fibroblasts by partially activating p53 signaling
Source: Cell Death Dis. 2021 Jan 5;12(1):40. doi: 10.1038/s41419-020-03323-x (PMC7791133; doi:10.1038/s41419-020-03323-x)

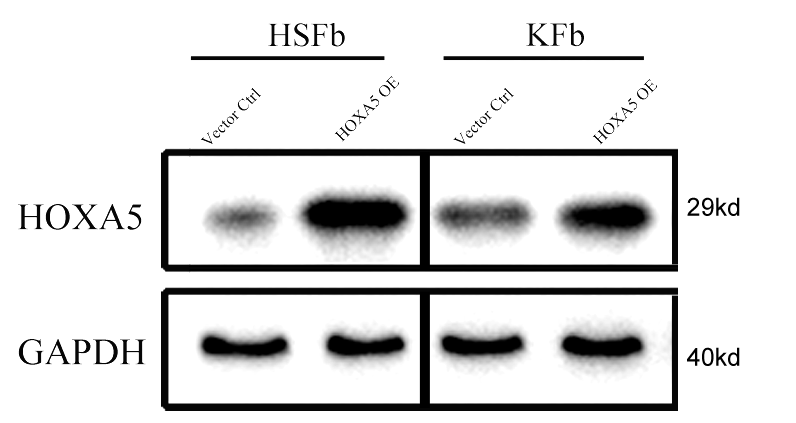

Supplement: Supplementary file 2 — Supplemental figure 1 [file 41419_2020_3323_MOESM2_ESM.tif]

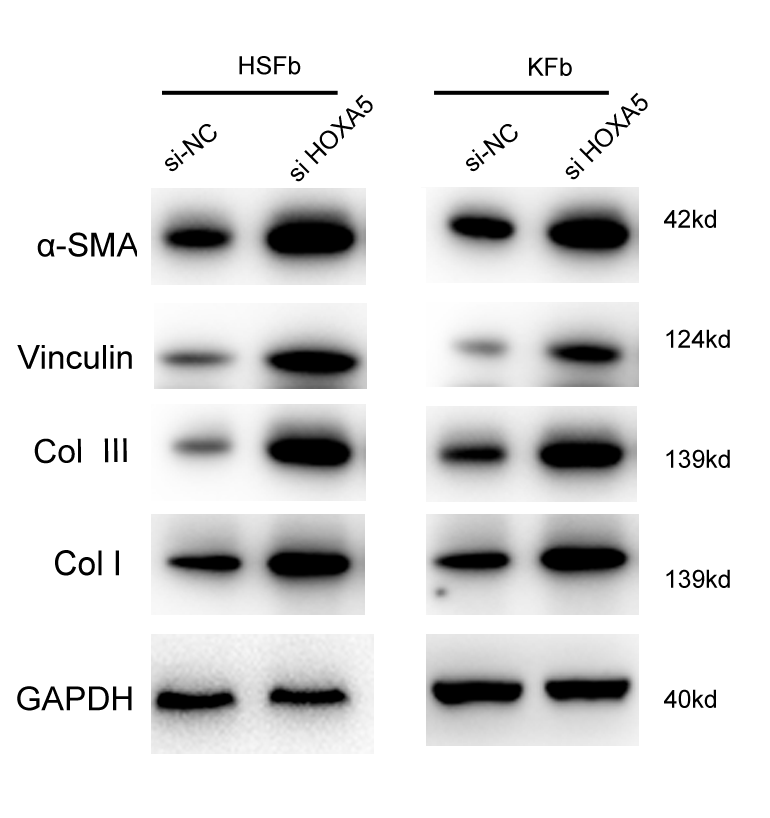

Supplement: Supplementary file 3 — Supplemental figure 2 [file 41419_2020_3323_MOESM3_ESM.tif]

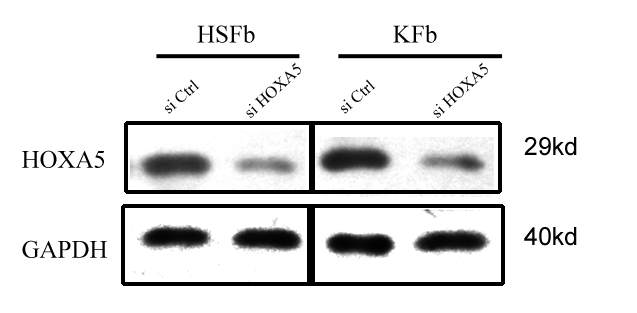

Supplement: Supplementary file 4 — Supplemental figure 3 [file 41419_2020_3323_MOESM4_ESM.tif]
